# Nanosphere Loaded With Curcumin Inhibits the Gastrointestinal Cell Death Signaling Pathway Induced by the Foodborne Pathogen *Vibrio vulnificus*

**DOI:** 10.3390/cells9030631

**Published:** 2020-03-05

**Authors:** Ji-Yun Kim, Young-Min Lee, Do-Wan Kim, Taesun Min, Sei-Jung Lee

**Affiliations:** 1 Department of Pharmaceutical Engineering, Daegu Haany University, Gyeongsan 38610, Korea; 2 Department of Animal Biotechnology, Faculty of Biotechnology, SARI, Jeju National University, Jeju 63243, Korea

**Keywords:** apoptosis, curcumin nanosphere, gastrointestinal cell death, *Vibrio vulnificus*, VvhA

## Abstract

Curcumin, a hydrophobic polyphenol of turmeric, has a variety of biological functions as a herbal supplement, but its poor gastric absorption rate is one of the major factors limiting its oral bioavailability. In the present study, we have investigated the functional role of a nanosphere loaded with curcumin (CN) during host cell death elicited by the Gram-negative bacterium *V. vulnificus* in human gastrointestinal epithelial HT-29 cells and an ileal-ligated mouse model. The recombinant protein (r) VvhA produced by *V. vulnificus* significantly reduced the viability of HT-29 cells. The cytotoxic effect of rVvhA was restored upon a treatment with CN (100 ng/mL), which had shown 1000-fold higher anti-apoptotic efficacy than curcumin. CN inhibited the phosphorylation of c-Src and PKC mediated by intracellular ROS responsible for the distinctive activation of the MAPKs in rVvhA-treated HT-29 cells. Interestingly, CN significantly restored the expression of Bax, Bcl-2, and cleaved caspase-3 as regulated by the phosphorylation of NF-κB. In mouse models of *V. vulnificus* infection, treatment with CN had a blocking effect that elevated the levels of TUNEL-positive DNA fragmentation and apoptosis-related proteins. These results indicate that CN is a functional agent that manipulates the *V. vulnificus* VvhA signaling pathway responsible for gastrointestinal cell death.

## 1. Introduction

*Vibrio (V.) vulnificus* is a pathogenic marine bacterium associated with foodborne illnesses, often causing gastroenteritis, septicemia, and diarrhea [1]. Infection with *V. vulnificus* is cytotoxic to host cells, and its virulence is mediated by secreted cytotoxins and enzymes, such as VvhA, MARTX, VvpE, and VvpM [1,2,3,4,5,6]. VvhA is one of the most potent pore-forming cytotoxins capable of killing mice at sub-μg levels, and it plays a crucial role in the pathogenesis and dissemination of *V. vulnificus* by facilitating intestinal paracellular permeabilization [2]. Recent studies have suggested that recombinant (r) VvhA stimulates the mitochondrial apoptotic machinery through the production of intracellular reactive oxygen species (ROS) derived from NADPH oxidase 2 (NOX2) located on membrane lipid rafts, thereby governing the PKC, MAPK, and NF-κB signaling pathways during the infection of host cells [7]. Specifically, rVvhA induces the formation of autophagosomes via the lipid raft-dependent c-Src/ROS signaling pathway in promoting intestinal epithelial cell death [2]. These results suggest that VvhA is responsible for the pathogenesis of *V. vulnificus* via the induction of host cell death, through which VvhA provokes the formation of ROS and distinctively manipulates various modes of intestinal epithelial cell death to facilitate bacterial dissemination and virulence effects. Thus, neutralizing the pathogenic signaling pathways triggered by rVvhA may offer potential therapeutic stratagems for foodborne illnesses caused by *V. vulnificus* infections.

Many studies have focused on the discovery and identification of safe new drugs against bacterial infections [8]. However, there are potential problems with therapeutic/suppressive agents when eliminating bacteria per se owing to the growing problem of antibiotic resistance. Thus, it is important to find good agents that manipulate the bacterial signaling pathway without antibiotic treatments for protection of the host cell. Curcumin, the principal active ingredient of *Curcuma longa* (Linn.), has traditionally been used as a remedy for the treatment of many diseases. Previous researchers reported that curcumin possesses a broad protective function for the host by modulating numerous molecular targets, such as growth factors, ROS, transcription factors, and apoptotic genes [9]. However, despite the enormous curative potential of curcumin, its therapeutic efficiency has been limited, partly due to its poor gastric absorption rate, low oral bioavailability, and its high hydrophobicity in the gut [10]. Recently, we developed a nanotechnology-based delivery system for curcumin which uses lecithin, a vegetable-based phospholipid that is a major component of all cell membranes [11]. This active nanosphere, when loaded with curcumin, designated as CN, has the ability to improve the aqueous-phase solubility and bioavailability, showing many biological functions in gastrointestinal epithelial cells and in the mouse gut [12,13]. While CN has been shown to have attractive therapeutic efficiency in gastrointestinal diseases, its role in the pathogenesis of a Gram-negative *V. vulnificus* infection remains unclear. Thus, in this study, we investigated the functional role of a nanosphere loaded with curcumin (CN) during host cell death elicited by the foodborne pathogen *V. vulnificus* in human gastrointestinal epithelial HT-29 cells and an ileal-ligated mouse model.

## 2. Materials and Methods

### 2.1. Chemicals

*Curcuma Longa* Linn (powdered form) and lecithin (L-α-phosphatidylcholine) were obtained from Sigma-Aldrich (St. Louis, MO, USA). The organic solvents such as toluene and dichloromethane were purchased from Fisher Scientific (Waltham, MA, USA). Fetal bovine serum (FBS) and phosphate-buffered saline (PBS) were purchased from GE Healthcare (Logan, UT, USA). The following antibodies were obtained: c-Src, phospho-c-Src, PKC, phospho-PKC, JNK, phospho-JNK, p38, phospho-p38, ERK, phospho-ERK, IκBα, phospho-IκBα, NF-κBp65, phospho-NF-κBp65, Bcl-2, Bax, cleaved caspase-3, and β-actin antibodies (Santa Cruz Biotechnology, Paso Robles, CA, USA); The following reagents were obtained: N-acetylcysteine (NAC) (Tocris, KOMA Biotech, Seoul, Korea) and 5-(and-6)-chloromethyl-2′,7′- dichlorodihydrofluorescein diacetate, acetyl ester (CM-H_2_DCFDA) (Invitrogen, Carlsbad, CA, USA). PP2, SP600125, Bisindolylmaleimide I, and Bay11-7082 were obtained from MedChemExpress (Monmouth Junction, NJ, USA). All other reagents did not show any critical cytotoxic effects by themselves.

### 2.2. Cells

Human gastrointestinal epithelial HT-29 cells were obtained from the Korean Cell Line Bank (KCLB, Seoul, Korea). HT-29 cells were cultured at 37 °C in 5% CO_2_ in dulbecco’s modified eagle’s medium (DMEM; GE Healthcare, Logan, UT, USA) with 10% FBS and antibiotics. The medium was renewed twice a week. HT-29 cells have previously been used to study the apoptotic process induced by *V. vulnificus* due to their physiologically relevant characteristics responsible for the adhesion and invasion of pathogens [14].

### 2.3. Purification of the Recombinant Protein (r)VvhA

To test the pathophysiological role of VvhA in HT-29 cells, we purified a recombinant protein of VvhA (rVvhA) from *V. vulnificus* as previously described [2]. Briefly, the conserved region of the VvhA gene sequence was amplified and then cloned into a pET29a (+) vector (Novagen, Madison, WI, USA) to generate the pKS1201 (Table 1). To induce the protein expression, *E. coli* BL21 (DE3) harboring the pKS1201 was grown in LB media with ampicillin. The cells mixed with Ni-NTA agarose beads (Qiagen, Valencia, CA, USA) were loaded on Bio-Spin Chromatography Columns (Bio-Rad Laboratories, Hercules, CA, USA). The eluted VvhA protein was dialyzed with Slide-A-Lyzer Dialysis Cassettes (Thermo Scientific, Hudson, NH, USA) and assessed the level of endotoxin in the purified rVvhA by using Chromogenic Endotoxin Quantitation kit (Thermo Fisher Scientific Inc., Waltham, MA, USA).

### 2.4. Preparation of Curcumin Nanosphere (CN)

We developed the curcumin nanosphere (CN) loaded with curcumin as previously described [12]. Briefly, curcumin (5 mg/mL) dissolved in 20 mL of toluene was added dropwise to the boiling water (50 mL) under continuous ultrasonication with a frequency of 50 kHz and stirred at 1000× *g* for 20 min followed by concentration under reduced pressure at 40 °C using a rotary evaporator. The samples were collected and lyophilized to obtain curcumin nanoparticles (CP). A lecithin mixture consisting of lecithin (0.2 mg) and dichloromethane (40 µL) was mixed with the curcumin nanoparticles in a ratio of 1:1 under constant stirring. The mixture was placed in an ultrasonicator for 2 h at 20–30 kHz to obtain a clear orange-colored solution, which is designated as curcumin nanosphere (CN). The CN made up of the primary CP was dried with a freeze dryer (Sam Won, Seoul, Korea) and stored at −70 °C.

### 2.5. Ultraviolet-Visible Spectroscopy (UV-Vis) Analysis

The characteristic peaks of the CN solution were determined using a Ultraviolet-visible (UV-Vis) spectrophotometer (SPARK, Seestrasse, Männedorf, Switzerland) at the range of 300 to 700 nm wavelengths.

### 2.6. Field Emission Scanning Electron Microscope (FE-SEM) Measurement

The surface features of synthesized CN were monitored by using the Field emission scanning electron microscope (FE-SEM, JSM-6700F, JEOL Ltd., Seoul, Korea). The aqueous dispersion of CN was spread over a silicon wafer and dried under atmospheric air. The samples were placed in carbon stubs and then coated with a 200-Å-thick gold-palladium layer under vacuum conditions. The spectra of CN showed spherical and uniform shapes with a diameter below 100 nm.

### 2.7. Fourier-Transform Infrared Spectroscopy (FT-IR) Measurement

The chemical structures of the CN were investigated by using a Fourier-transform infrared spectroscopy (FT-IR, PerkinElmer Frontier IR/NIR systems, Waltham, MA, USA). Their spectra were recorded in a range between 500–4000 cm^−1^ wavelength with a resolution range of 4 cm^−1^ and 64 scans.

### 2.8. Cell Viability Assay

A cell viability assay was conducted using the EZ-CYTOX cell viability kit (Dail-Lab Service, Seoul, Korea) according to the manufacturer’s instructions. Cells were cultured on 96-well culture plates. After incubation with rVvhA and CN, 10 μL of EZ-CYTOX master mix was added to each well for 1 h. Cell viability was analyzed by measuring the absorbance at 450 nm.

### 2.9. Cell Number Count

To determine the total number of cells, cells were trypsinized from the cell culture dishes. The cell suspension was stained with a 0.4% (*w/v*) trypan blue, and the total number of live cells was counted using a hemocytometer. Cells unable to exclude the dye were considered nonviable.

### 2.10. Reactive Oxygen Species (ROS) Detection

The production of intracellular ROS was determined by using CM-H_2_DCFDA. Cells treated with CM-H_2_DCFDA were washed twice with PBS and then scraped. A 100 μL of cell suspension was loaded into 96-well culture plates and detected by a fluorescent microplate reader (SPARK, Seestrasse, Männedorf, Switzerland) between an excitation and emission wavelengths (485 and 535 nm) respectively.

### 2.11. Immunofluorescence Analysis

HT-29 cells were fixed in 4% paraformaldehyde for 10 min, permeabilized in 0.1% Triton X-100 for 5 min, and blocked in 5% (*v/v*) normal goat serum (NGS) for 30 min. Cells were then stained with primary antibody at overnight 4 °C. Following rinses with PBS, the cells were incubated with Alexa 488-conjugated goat anti-rabbit and anti-mouse IgM (Invitrogen Co., Carlsbad, CA, USA), and counterstained with 4′,6-diamidino-2-phenylindole (DAPI) in 5% (*v/v*) NGS for 2 h. After washing with PBS, the samples were mounted on slides and visualized with an Olympus FluoView™ 300 confocal microscope (Olympus America Inc., Center Valley, PA, USA) with 400× objective.

### 2.12. Western Blot Analysis

Cells were harvested, washed with PBS, and lysed with RIPA lysis buffer (ATTO Corp., Tokyo, Japan) for 30 min on ice. Protein concentrations were determined by BCA Protein Assay kits (Pierce, Rockford, IL, USA). Equal amounts of protein (20 μg) were resolved by 8–12% sodium dodecyl sulfate polyacrylamide gel electrophoresis (SDS-PAGE) and transferred to PVDF membranes. The PVDF membranes were washed with TBST solution (Tween-20 (0.05%), 10 mM Tris-HCl (pH 7.6), and 150 mM NaCl), blocked with skim milk (5%) for 30 min and incubated with the appropriate primary antibody at 4 °C overnight. Each membrane was then incubated with an HRP-conjugated secondary antibody for 2 h. The bands were detected by using the Bio-Rad Chemi Doc™ XRS + System (Bio-Rad, Hercules, CA, USA). The intensity of bands was quantified using Scion imaging software (Scion Image Beta 4.02, Frederick, MD, USA).

### 2.13. Ileal-Ligated Mouse Model

All animal procedures were performed following the National Institutes of Health Guidelines for the Humane Treatment of Animals, with approval from the Institutional Animal Care and Use Committee of Daegu Hanny University (DHU-2020-001). Seven-week-old mice (*n* = 6) were anesthetized under ether and placed on a warm pad to maintain body temperature at 37 °C. A small abdominal incision was made to exteriorize the ileal region of small intestine, which was followed by tying both ends of the ileum by silk suture. The closed loops were then instilled with 100 µL of PBS alone, PBS containing rVvhA or PBS containing rVvhA and CN (10^−2^ mg/kg). The small abdominal incision were closed with surgical sutures for 2 h. Mice were sacrificed, and the intestinal loops were removed for the western blot analysis and the terminal deoxynucleotidyl transferase dUTP nick-end labeling (TUNEL) assay.

### 2.14. Apoptosis Detection

The apoptotic cells in ileal tissue were also detected by a terminal deoxynucleotidyl transferase dUTP nick-end labeling (TUNEL) assay using an apoptosis detection kit (Promega, Seoul, Korea) according to the manufacturer’s instructions. Intestinal sections were prepared as described above. The tissue sections were stripped from proteins by incubation with 100 µL of proteinase K for 10 min and washed three times in PBS for 5 min respectively. The sections were covered with recombinant terminal deoxynucleotidyl transferase (rTDT) reaction mix at 37 °C for 60 min. After the sections were rinsed with PBS, the apoptotic cells were visualized with an Olympus FluoView™ 300 confocal microscope.

### 2.15. Flow Cytometry

Cells were synchronized in the G0/G1 phase by culture in serum-free medium for 24 h before incubation with melatonin and rVvhA. Necrotic cell death was detected with an Annexin V conjugated with FITC and PI staining kit (BD Biosciences, Franklin Lakes, NJ, USA) according to the manufacturer’s instructions. Briefly, the cells were detached with 0.05% trypsin/EDTA, and 1 × 10^5^ cells were resuspended with Annexin V binding buffer. The cells were then stained with FITC-Annexin V conjugate (25 μg/mL) and PI (125 ng/mL) and incubated for 15 min at room temperature in the dark. The sample was read by performing flow cytometry (CytoFLEX V0-B2-R2, Beckman Coulter, Fullerton, CA, USA). Samples were gated to exclude debris (forward light scatter [FSC] area versus side scatter area), and cell doublets were excluded by applying FSC-area versus FSC-width analysis. Samples were analyzed by using Flowing software (developed by Perttu Terho, Turku, Finland). AnnexinV-negative/PI-positive (Q1) cells were considered necrotic, AnnexinV-positive and PI-negative (Q2) cells were considered early apoptotic, AnnexinV-negative and PI-negative (Q3) cells were considered viable, and AnnexinV-positive and PI-positive (Q4) cells were considered early and late apoptotic. To calculate the percentage of total apoptotic cells, the following formula was used: Apoptotic cells (%) = Q1 + Q2 + Q4.

### 2.16. Solubility Analysis

The entire procedure was carried out in a room conditioned at 25 °C. The solubility was tested in deionized water and in ethanol (99.5%). The samples were added in quantities sufficient to reach excess the content of curcumin and CN in the solvent exceeding the saturation point. The stirred samples were further taken in test tubes and centrifuged at 10,000× *g* for 15 min. The supernants were collected and filtered through 0.22 μm nylon membrane filter (Thermo Scientific, Hudson, NH, USA). Absorbance was measured at 430 nm using a UV-Vis spectrophotometer.

### 2.17. Statistical Analysis

Results are expressed as means ± standard errors (S.E.). All experiments were analyzed by ANOVA, followed in some cases by a comparison of treatment means with control using the Bonferroni–Dunn test. Differences were considered statistically significant at *p* < 0.05.

## 3. Results

### 3.1. Characterization of the Curcumin Nanosphere (CN)

**Figure 1:**
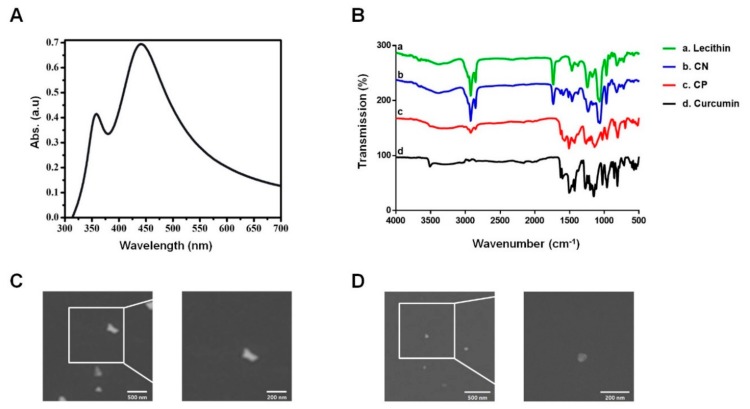
Characterization of curcumin nanosphere (CN). (**A**) Ultraviolet-visible (UV-Vis) spectrum analysis of CN. (**B**) Fourier-transform infrared spectroscopy (FT-IR) analysis of CN. (**A**) Lecithin. (**B**) Curcumin nanosphere. (**C**) Curcumin nanoparticle. (**D**) Curcumin. (**C**) Field emission scanning electron microscope (FE-SEM) analysis of curcumin nanoparticle (CP). (**D**) FE-SEM analysis of CN. *n* = 3.

We previously reported that CN loaded with curcumin has the ability to improve aqueous-phase solubility and bioavailability levels [12]. To characterize the curcumin nanosphere (CN) further, UV-Vis spectrophotometry was used to measure its characteristic absorption peak. The UV-Vis spectra revealed a sharp absorption peak around the wavelength of 430 nm, known to be the signature peak of curcumin (Figure 1A). Fourier-transform infrared spectroscopy (FT-IR) was used to identify variations in the functional groups in CN. Curcumin showed its signature peaks at 3508 cm^−1^ (phenolic O-H stretching), 1626 cm^−1^ (aromatic moiety C=C stretching), 1599 cm^−1^ (benzene ring stretching), 1502 cm^−1^ (aromatic C=C stretching), 1426 cm^−1^ (olefinic C-H bending), 1272 cm^−1^ (aromatic C-O stretching), and 1025 cm^−1^ (C-O-C stretching). The curcumin nanoparticle (CP) exhibited peaks similar to those of the curcumin sample at 3501 cm^−1^ (phenolic O-H stretching), 1624 cm^−1^ (aromatic moiety C=C stretching), 1568 cm^−1^ (benzene ring stretching), 1507 cm^−1^ (aromatic C=C stretching), 1426 cm^−1^ (olefinic C-H bending), 1260 cm^−1^ (aromatic C-O stretching), and 1025 cm^−1^ (C-O-C stretching). When curcumin was incorporated in lecithin (CN), the resulting typical peaks and shapes were similar to those of lecithin at 1735 cm^−1^ (esters C=O stretch), 1241 cm^−1^ (phosphorus oxide bond), 1085 cm^−1^ and 1064 cm^−1^ (aliphatic amine C-*n* stretch). Importantly, the FT-IR absorption spectra were found at 1628 cm^−1^ (aromatic moiety C=C stretching), 1589 cm^−1^ (benzene ring stretching), and 1514 cm^−1^ (aromatic C=C stretching), which are the characteristic peaks of curcumin, indicating the existence of curcumin in the lecithin. The FT-IR absorption spectra of CN were different at 1502~1455 cm^−1^ and 1231~1063 cm^−1^, compared to curcumin, suggesting that there are interactions between curcumin and lecithin [15]. The missing peak at 3508 cm^−1^ in the CN indicates the interaction of the phenolic-OH of curcumin with the lecithin, most likely through hydrogen bonding and polar interactions (Figure 1B). In addition, we attempted to compare the surface features of CN with those of CP using the FE-SEM. We found that CN with diameters less than 100 nm were spherical in shape (Figure 1C), whereas the CP had different shapes, such as hexagonal, polygonal and triangular (Figure 1D). These results indicate that the shape of the CN can be modulated by controlling the morphology of the primary CP to tailor the physical and chemical properties.

### 3.2. CN Has An Inhibitory Effect on the Production of ROS Responsible for Cytotoxicity Caused by V. Vulnificus, VvhA

**Figure 2:**
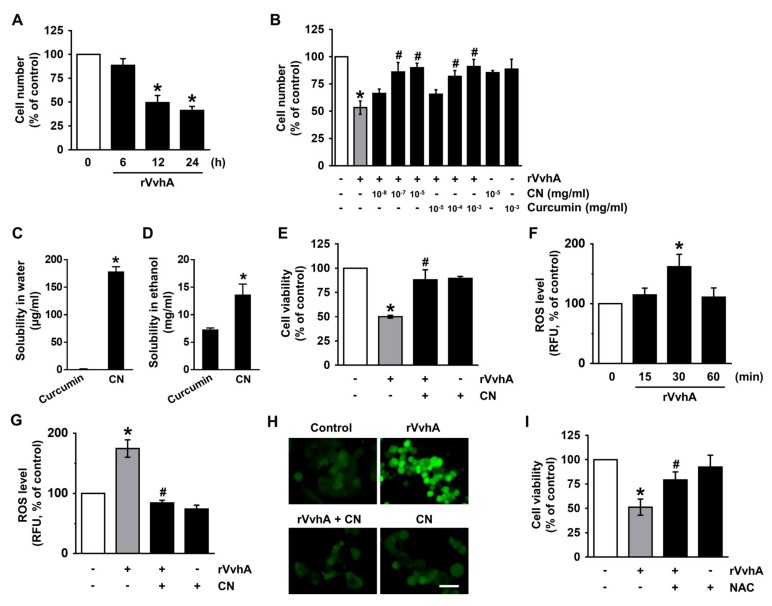
CN has an inhibitory effect on the production of reactive oxygen species (ROS) responsible for cytotoxicity caused by *V. vulnificus*, VvhA. (**A**) Time responses of cell viability in cell treated with rVvhA is shown. *n* = 4. * *p* ≤ 0.01 vs. 0 h. (**B**) Cells were treated with CN (10^−8^~10^−5^ mg/mL) or curcumin (10^−5^~10^−3^ mg/mL) in the presence of rVvhA for 24 h. The number of cells was determined by cell counting assay. *n* = 4. * *p* ≤ 0.01 vs. control. # *p* ≤ 0.05 vs. rVvhA alone. The solubility of CN in water (**C**) and ethanol (**D**) is shown. *n* = 5. * *p* ≤ 0.01 vs. Curcumin (**E**) Cells were treated with CN (10^−7^ mg/mL) and rVvhA for 24 h. Cell viability was determined by EZ-CYTOX kit. *n* = 3. * *p* ≤ 0.001 vs. control. # *p* ≤ 0.01 vs. rVvhA alone. (**F**) The level of ROS in rVvhA-treated cell is shown. *n* = 3. * *p* ≤ 0.01 vs. 0 min. (**G**) Cells were incubated with CN and rVvhA for 30 min. The level of ROS production is shown. *n* = 3. * *p* ≤ 0.01 vs. control. # *p* ≤ 0.001 vs. rVvhA alone. RFU, Relative fluorescence units. (**H**) ROS production (green) was visualized by confocal microscopy. Scale bars, 100 μm (Original magnification ×100). *n* = 3. (**I**) Cells were pretreated with NAC (10 μM) for 30 min prior exposure to rVvhA for 24 h. *n* = 4. * *p* ≤ 0.001 vs. control. # *p* ≤ 0.05 vs. rVvhA alone. Data represent means ± S.E.

Curcumin has a variety of biological functions, but it has been reported that the high hydrophobicity of curcumin in the gastrointestinal epithelium limits its bioavailability. To determine the functional role of CN loaded with curcumin in the gastrointestinal epithelium, we used human gastrointestinal epithelial HT-29 cells, which form a homogeneous population of polarized epithelial cells. HT-29 cells were exposed to the recombinant protein (r) VvhA produced by *V. vulnificus* for 24 h. A significant decrease in the cell number was observed starting 12 h after incubation with 50 pg/mL of rVvhA (Figure 2A). The cytotoxic effect of rVvhA decreased upon treatment with CN (from 10^−7^ mg/mL). On the other hand, a recovery effect of curcumin on the cytotoxicity was observed starting at an amount of 10^−4^ mg/mL. These results indicate that CN has greater protective efficacy by 1000-fold against *V. vulnificus* infection than curcumin (Figure 2B). The discrepancy with regard to the bioactivity of CN may be due to differences in the solubility. In this context, we have tried to do an additional experiment comparing the solubility of CN with those of curcumin using a spectrophotometric method. As shown in Figure 2C, the water solubility of CN was 179 μg/mL which is about 160 times higher than the curcumin. This result indicates that the use of nanosuspensions has evident potential as dispersing agent of curcumin in aqueous formulations. The solubility of CN and curcumin in ethanol was much higher than in water. However, the ethanol solubility of CN was around 1.5 times higher than the curcumin (Figure 2D). This means that the bioactivity of CN is improved by reducing the particles size. We further confirmed the pharmacological effect of CN against *V. vulnificus* infection through a cell viability assay based on mitochondrial respiration. In contrast to the control, 50 pg/mL of rVvhA reduced the cell viability for 12 h (Figure 2E), with this decrease significantly blocked by a treatment with 100 pg/mL (10^−7^ mg/mL) of CN. To determine the molecular mechanisms related to host cell protection, we conducted a closer examination of the roles of CN in the production of reactive oxygen species (ROS), which are crucial for manipulating the bacterial signaling pathway. A significant increase in the ROS level was observed at 30 min after incubation with 50 pg/mL of rVvhA (Figure 2F), though the increase could be blocked by 100 pg/mL of CN (Figure 2G). The anti-oxidative effect of CN was further visualized by staining HT-29 cells with a fluorescent dye, CM-H_2_DCFDA (Figure 2H). The cytotoxic effect of rVvhA was significantly blocked by the treatment with an antioxidant, N-acetylcysteine (NAC) (Figure 2I). These results indicate that the host protective effect of CN is mediated by its anti-oxidative activity against *V. vulnificus* infection.

### 3.3. CN Regulates the Activation of c-Src and PKC Induced by rVvhA

**Figure 3:**
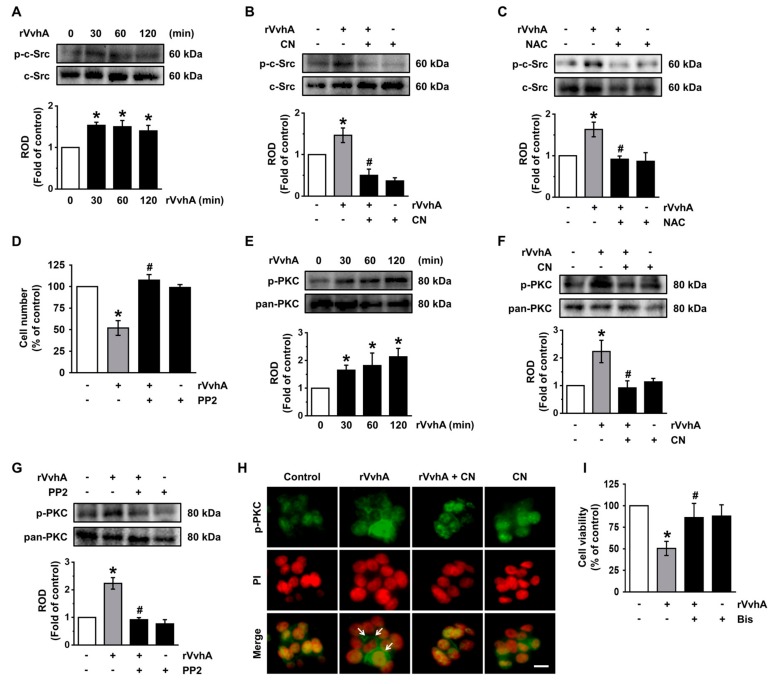
CN regulates the activation of c-Src and PKC induced by rVvhA. (**A**) Time responses of phosphorylation of c-Src in cells exposed to rVvhA is shown. *n* = 3. * *p* ≤ 0.05 vs. control. (**B**) The inhibitory effect of CN on phosphorylation of c-Src in rVvhA-treated cells is shown. *n* = 3. * *p* ≤ 0.05 vs. control. # *p* ≤ 0.01 vs. rVvhA alone. (**C**) Cells were pretreated with NAC prior exposure to rVvhA for 30 min. *n* = 3. * *p* ≤ 0.05 vs. control. # *p* ≤ 0.01 vs. rVvhA alone. (**D**) Cells were pretreated with PP2 (10 μM) for 30 min prior exposure to rVvhA for 24 h. Cell viability was determined by cell counting assay. *n* = 4. * *p* ≤ 0.01 vs. control. # *p* ≤ 0.001 vs. rVvhA alone. (**E**) Phosphorylation of PKC in cells exposed to rVvhA is confirmed by western blot. *n* = 3. * *p* ≤ 0.05 vs. control. (**F**) The inhibitory effect of CN on phosphorylation of PKC in rVvhA-treated cells is shown. *n* = 3. * *p* ≤ 0.05 vs. control. # *p* ≤ 0.01 vs. rVvhA alone. (**G**) Cells were pretreated with PP2 prior exposure to rVvhA for 30 min. *n* = 3. * *p* ≤ 0.01 vs. control. # *p* ≤ 0.001 vs. rVvhA alone. (**H**) Cells were treated with CN and rVvhA to confirm the membrane translocation of PKC. Scale bars, 100 μm (Original magnification × 400). *n* = 3. (**I**) Cells were pretreated with Bisindolylmaleimide I (10 μM) were incubated with rVvhA for 24 h. *n* = 4. * *p* ≤ 0.001 vs. control. # *p* ≤ 0.01 vs. rVvhA alone. Data represent the mean ± S.E. (**A**–**C** and **E**–**G**) ROD is the abbreviation for relative optical density.

ROS have been shown to regulate the activity of the c-Src tyrosine kinase, which acts as a hub linking signals between the inner and outer environments of host cells during a bacterial infection [2,16]. A significant increase in the level of c-Src phosphorylation was observed at 30 min after incubation with 50 pg/mL of rVvhA (Figure 3A), though the increase at 30 min could be inhibited by CN (Figure 3B) as well as NAC (Figure 3C). Moreover, a c-Src inhibitor, PP2, significantly inhibited cell death induced by rVvhA (Figure 3D). These results indicate that CN has the ability to inhibit the phosphorylation of c-Src mediated by ROS during cell death induced by *V. vulnificus* infection. Activation of protein kinase C (PKC) could be induced by c-Src, and this event typically influences the cellular signal transduction process for bacterial entry [17,18]. In contrast to the control, 50 pg/mL of rVvhA induced the phosphorylation of PKC for 120 min (Figure 3E), with this increase at 60 min significantly blocked by a treatment with CN (Figure 3F) and by PP2 (Figure 3G). The inhibitory effect of CN on the membrane translocation of PKC induced by rVvhA was further confirmed by the immunofluorescence staining of pan-PKC (Figure 3H). Interestingly, a PKC inhibitor, Bisindolylmaleimide I, inhibited the cell death caused by rVvhA (Figure 3I). These results suggest that CN inhibits the activation of c-Src and PKC induced by rVvhA and that these pharmacological effects of CN are critical for cell protection in the event of a *V. vulnificus* infection.

### 3.4. CN Uniquely Regulates the JNK/NF-κB Pathway Responsible for Cell Death Caused by rVvhA

**Figure 4:**
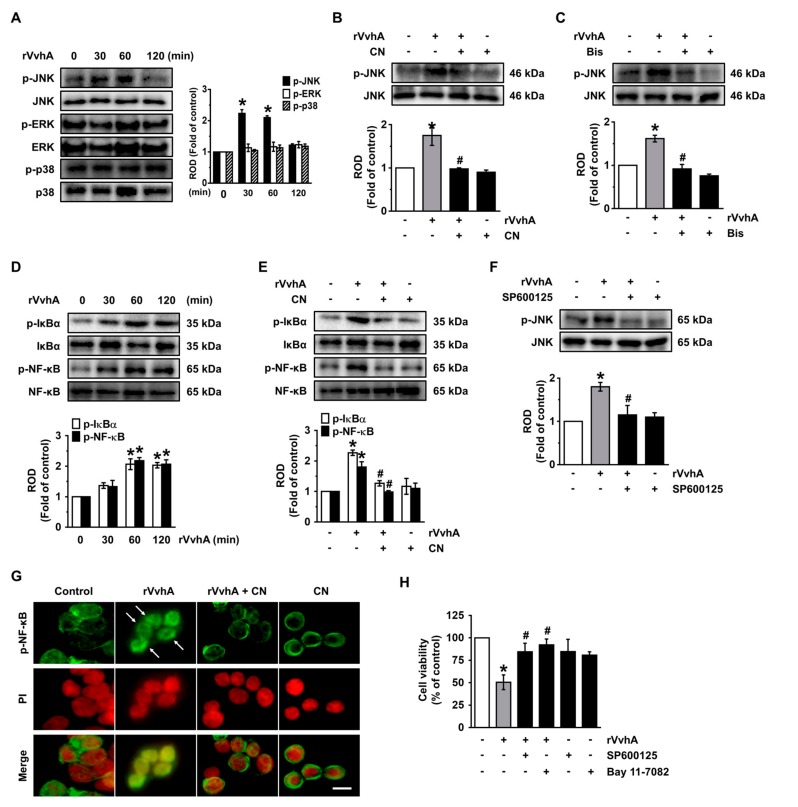
CN uniquely regulates the c-Jun n-terminal kinase (JNK)/nuclear factor-kappa B (NF-κB) pathway responsible for cell death caused by rVvhA. (**A**) Time responses of phosphorylation of mitogen-activated protein kinases (MAPK) in cells exposed to rVvhA are shown. *n* = 3. * *p* ≤ 0.001 vs. control. (**B**) The effect of CN on the phosphorylation of JNK is shown. *n* = 3. * *p* ≤ 0.01 vs. control. # *p* ≤ 0.01 vs. rVvhA alone. (**C**) Cells were pretreated with Bisindolylmaleimide I prior exposure to rVvhA for 30 min. (**D**) Cells pretreated with SP600125 (10 μM) were incubated with rVvhA for 24 h. *n* = 3. * *p* ≤ 0.001 vs. control. # *p* ≤ 0.01 vs. rVvhA alone. (**E**) Time responses of phosphorylation of IκBα and NF-κB in cells exposed to rVvhA are shown. *n* = 3. * *p* ≤ 0.001 vs. control. (**F**) The effect of CN on the phosphorylation of IκBα and NF-κB is shown. *n* = 3. * *p* ≤ 0.01 vs. control. # *p* ≤ 0.01 vs. rVvhA alone. (**G**) Cells were treated with CN and rVvhA to confirm the nuclear translocalization of NF-κB determined by immunofluorescence staining. Scale bars, 100 μm (Original magnification × 400). *n* = 3. (**H**) Cells were pretreated with SP600125 and Bay 11-7082 (10 μM) prior exposure to rVvhA for 30 min. *n* = 3. * *p* ≤ 0.001 vs. control. # *p* ≤ 0.01 vs. rVvhA alone. Data represent the mean ± S.E. (**A**–**F**) ROD is the abbreviation for relative optical density.

We then determined how rVvhA is linked to the activation of mitogen-activated protein kinases (MAPKs) and transcription factor NF-κB, which are interesting downstream mediators of PKC during the regulation of host signaling pathways infected by many bacterial pathogens [19,20]. rVvhA increased the phosphorylation of JNK between 30 and 60 min, but it did not affect the activation of either ERK or p38 MAPK (Figure 4A). However, the unique activation of JNK at 60 min was significantly inhibited by a treatment with CN (Figure 4B) and by a PKC inhibitor (Figure 4C). The degradation of IκBα by its phosphorylation leads to the release and translocation of NF-κB into the nucleus and the subsequent activation of several target genes [21]. Significant increases in the phosphorylation outcomes of IκBα and NF-κB were observed 30 and 60 min after incubation with 50 pg/mL of rVvhA (Figure 4D), though the increase at 60 min could be blocked by 100 pg/mL of CN (Figure 4E) and by the JNK inhibito, SP600125 (Figure 4F). The inhibitory effect of CN on the nucleic activation of p-NF-κB induced by rVvhA was also confirmed by the immunofluorescence method (Figure 4G). The cytotoxic effect of rVvhA was significantly blocked by a treatment with either SP600125 or the NF-κB inhibitor, Bay 11-7082 (Figure 4H). These results suggest that CN inhibits the JNK-mediated activation of NF-κB, which is required for the signaling pathway evoked by rVvhA during the promotion of cell death.

### 3.5. The Role of CN on Apoptotic Cell Death Induced by rVvhA

**Figure 5:**
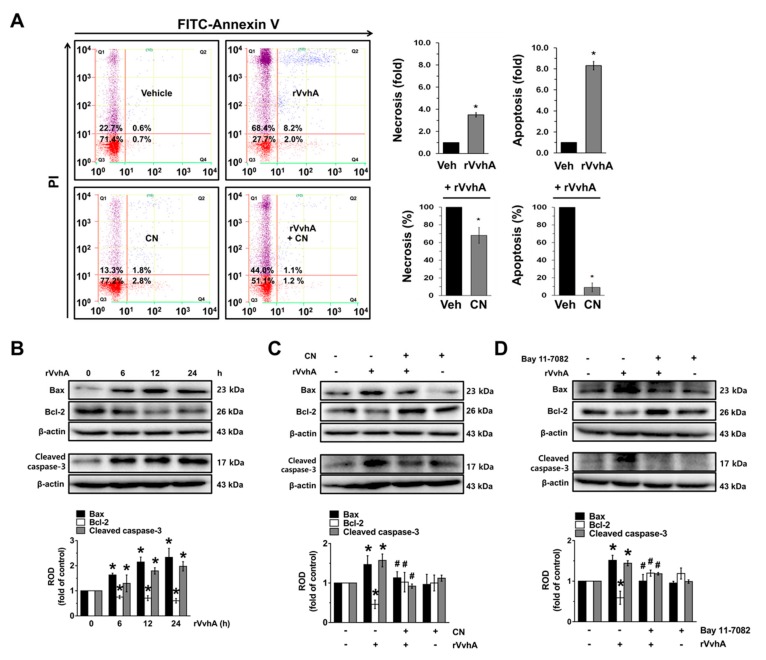
The role of CN on apoptotic cell death induced by rVvhA. (**A**) Cells were incubated with CN for 30 min prior to rVvhA exposure for 24 h. Percentages of total apoptotic cells were measured by using PI/Annexin V staining and flow cytometry. *n* = 4. * *p* ≤ 0.01 vs. control. # *p* ≤ 0.01 vs. rVvhA alone. (**B**) Expression of Bax, Bcl-2, and cleaved caspase-3 in cells exposed to rVvhA are shown. *n* = 3. * *p* ≤ 0.01 vs. control. (**C**) The effect of CN on the expression of apoptosis-related protein is shown. *n* = 3. * *p* ≤ 0.01 vs. control. # *p* ≤ 0.01 vs. rVvhA alone. (**D**) Cells were pretreated with Bay 11-7082 prior exposure to rVvhA for 24 h. *n* = 3. * *p* ≤ 0.001 vs. control. # *p* ≤ 0.01 vs. rVvhA alone. Data represent the mean ± S.E. (**B**–**D**) ROD is the abbreviation for relative optical density.

We additionally confirmed the pharmacological effect of CN against *V. vulnificus* infection using flow cytometric analyses to measure all aspects of cell death. rVvhA significantly stimulated the apoptosis (a 8.3 ± 0.4-fold increase compared to the control) of HT-29 cells, whereas with regard to necrosis, it had a minor effect (a 3.2 ± 0.2-fold increase compared to the control) (Figure 5A). In addition, we found that CN has greater inhibitory potency on apoptotic cell death as compared to necrotic cell death. The rVvhA increased the expression of cleaved caspase-3 and Bcl-2-associated X protein (Bax) but decreased Bcl-2 (Figure 5B). The stimulatory effect of rVvhA on the expression of mitochondria-mediated apoptotic factors was significantly blocked by a treatment with CN (Figure 5C) as well as Bay 11–7082 (Figure 5D). These results indicate that CN inhibits the mitochondrial apoptotic pathway through the inhibition of NF-κB signaling pathways in rVvhA-treated HT-29 cells.

### 3.6. CN Functionally Blocks Apoptotic Responses Caused by rVvhA in Mice

**Figure 6:**
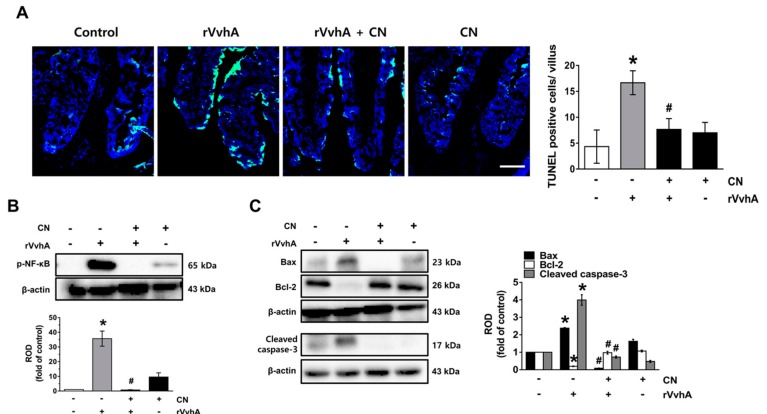
CN functionally blocks apoptotic responses caused by rVvhA in the mouse. The functional role of CN in apoptotic responses caused by rVvhA in the mouse ileum were evaluated in ileal-ligated mouse model after treatments with CN (10^−2^ mg/kg) and rVvhA. (**A**) The apoptotic cells (green) in frozen sections were detected by TUNEL assay using an apoptosis detection kit. Quantification of TUNEL-positive cells (right) are shown. *n* = 6. * *p* ≤ 0.001 vs. control. # *p* ≤ 0.001 vs. rVvhA alone. The level of NF-κB (**B**), Bax/Bcl-2 and cleaved caspase-3 (**C**) are shown. Data represent the mean ± S.E. *n* = 6. * *p* ≤ 0.001 vs. control. # *p* ≤ 0.001 vs. rVvhA alone. (**B**–**C**) ROD is the abbreviation for relative optical density.

To evaluate the clinical relevance of CN, we undertook additional experimentation using an ileal-ligated mouse model, in which a closed ileal loop was instilled with PBS (100 µL) containing rVvhA, rVvhA + CN, or CN for 2 h. In contrast to the control, rVvhA increased the number of TUNEL-positive cells, representing a characteristic hallmark of apoptosis, with this increase, observed at the apical region of the villi in the mouse ileum, significantly blocked by a treatment with CN (Figure 6A). A significant increase in the NF-κB phosphorylation (Figure 6B), a shift of the Bax/Bcl-2 ratio, and cleaved caspase-3 expression (Figure 6C) were observed after inoculation with rVvhA, though the increase at the mouse ileum could be inhibited by CN. These results suggest that CN functionally blocks apoptotic responses caused by rVvhA in the mouse ileum.

## 4. Discussion

Our data indicate that nanospheres loaded with curcumin (CN) neutralize the apoptotic signaling pathways triggered by *V. vulnificus* VvhA through the inhibition of c-Src/PKC/MAPKs/NF-κB activation occurring due to ROS production in human gastrointestinal epithelial HT-29 cells and an ileal-ligated mouse model. Therefore, our findings suggest that CN is a unique antibiotic-free agent that manipulates the foodborne pathogen signaling pathways caused by *V. vulnificus* infections.

Concerning the nano-formulation of curcumin, it has been shown that curcumin nanoparticles (CP) made up primary of curcumin improved bioavailability by more than three-fold [22,23]. Moreover, it was reported that shape control of CP with lecithin is a promising strategy as a feasible drug delivery system that may stabilize the physical and chemical properties of curcumin in terms of cell uptake, biodistribution, and accumulation at sites of interest [11]. In the present study, we have proven that CN and CP display similar FT-IR absorption spectra, which do not show shifts or losses of functional groups (aromatic moiety C=C stretching and benzene ring stretching) of the curcumin. In addition, the missing peak at 3508 cm^−1^ in the CN indicates the interaction of the phenolic-OH of curcumin with lecithin, most likely through hydrogen bonding and polar interactions [24]. These results suggest that CN is an active encapsulation product of CP, in which the naive curcumin efficiently interacts with the lecithin. Indeed, we have shown that the anti-apoptotic efficacy of CN was 1000-fold higher than that of the naive curcumin. We believe that the improved bioactivity of CN is in part due to the enhanced solubility of CN, which increases the surface of contact with the cell membrane. These findings hence suggest that CN is an effective drug-delivery system to improve the bioavailability of curcumin in the gut while also having an inhibitory effect on gastrointestinal apoptotic cell death induced by *V. vulnificus* VvhA.

VvhA is a critical toxin of *V. vulnificus* that possibly forms a redox signaling platform with NADPH oxidase 2 in the lipid rafts of the host cell membrane to amplify a variety of ROS-dependent signaling pathways during the regulation of apoptosis [25]. In the present study, we noted that CN at picogram levels has the functional ability to inhibit the production of the ROS responsible for cytotoxicity caused by VvhA. These results are similar to a result showing that melatonin, a well-known antioxidant, can block apoptotic and autophagic cell death occurring due to ROS production induced by VvhA [7], although the functional level of melatonin was in the microgram range. Thus, the activation of HT-29 cells with a low concentration of CN may offer a means by which to improve the potency of these cells without the need for an additional treatment of antibiotics to eliminate *V. vulnificus*.

We subsequently showed that CN has an inhibitory effect on the phosphorylation of c-Src responsible for PKC activation to block apoptotic cell death as induced by rVvhA. Many bacterial stimuli regulate the c-Src and PKC pathways, both of which are interesting candidates as downstream mediators of ROS [17,25]. c-Src, a non-receptor tyrosine kinase, is enriched in lipid rafts, where it plays a key role in the regulation of a diverse array of signaling events, including bacterial invasions. Indeed, the Src family has been shown to phosphorylate PKC on specific tyrosine residues [26]. Given the critical role of PKC in the promotion of the cell death process during *Enteropathogenic Escherichia (E.) coli* (EPEC), *C. perfringens*, and *V. vulnificus* infections [25,27,28], our current findings indicate that the CN inhibits the c-Src phosphorylation responsible for PKC activation to block apoptotic cell death as induced by rVvhA. These results are further supported by a previous study in which a bacterial infection of host cells causes acute damage to a variety of intracellular macromolecules via the rapid activation of c-Src, resulting in autophagic cell death [2], suggesting that c-Src is the signaling mediator during the molecular pathogenesis of bacterial infections [29]. Therefore, our findings suggest that CN has antioxidant abilities that inhibit the apoptotic signaling pathways evoked by rVvhA via the suppression of c-Src/PKC activation in cells.

To investigate the underlying molecular mechanisms of how the ROS/c-Src/PKC pathway is linked to the apoptotic cell death pathways, we focused on MAPKs/NF-κB pathways with regard to their possible roles in the promotion of cell death processes triggered by many bacterial stimuli [30]. Despite the frequent involvement of ERK and p38 MAPK in the ROS signaling pathway induced by a *H. pylori* infection [31], our results revealed that *V. vulnificus* VvhA uniquely regulates apoptotic cell death in these cases through the activation of the JNK-mediated NF-κB pathway. These results indicate that the cellular pathways activating JNK differ depending on the type of bacterial pathogen, though the activation of the host signal transduction caused by VvhA could be ameliorated by CN. Regarding the role of JNK in the activation of NF-κB, earlier work showed that the JNK pathway caused by ROS can influence the transcriptional activation of NF-κB in promoting apoptosis [32]. Indeed, NF-κB plays a critical role as a major transcriptional factor of the host apoptotic signaling pathway in the intestinal epithelium during bacterial pathogen infections [25,33]. In addition, a previous study has shown that increased NF-κB activity by JNK induces the transcription of the *bax* gene in response to butyric acid [34]. The mitochondrial translocation of Bax facilitates the release of mitochondrial cytochrome c as well as the binding of caspase-activating proteins to pro-caspase-9, necessary for the processing of caspase-3 [35,36]. Having shown that CN is able to inhibit a shift in the Bax/Bcl-2 ratio and cleaved caspase-3 activation through the inhibition of NF-κB activity in rVvhA-infected HT-29 cells, we suggest that CN regulates the distinctive infectious stratagems of *V. vulnificus* to control the apoptotic cell death pathway by manipulating the NF-κB pathway through the regulation of JNK. Based on these results, we suggest that CN has a potential role in the apoptotic cell death induced by rVvhA via the regulation of c-Src/PKC/JNK/NF-κB cascades. Interestingly, another type of pore-forming α-toxin released by *S. aureus* has been shown to induce massive levels of necrosis without having an apoptotic process [37], while EPEC was shown to disrupt the mitochondrial membrane potential, resulting in the release of cytochrome c and apoptosis [38]. Thus, these results imply that CN may not have a regulatory effect on *S. aureus*, whereas it has the unique function of being able to block the infectious mechanism caused by EPEC as well as the *V. vulnificus* pore-forming toxin in the prevention of the formation of the mitochondrial apoptotic pathway in the intestinal epithelium.

Finally, in the ileal-ligated mouse model treated with rVvhA, our results revealed that CN blocks apoptotic cell death mediated by DNA fragmentation, Bax, Bcl-2, and cleaved caspase-3, suggesting that the functional role of CN that neutralizes bacterial toxin activity involved in the host apoptotic pathway may provide potential therapeutic strategies for bacterial pathogen infections in the intestine, suggesting in turn that the functional role of CN of neutralizing the bacterial toxin activity involved in host apoptotic pathway may provide potential therapeutic strategies for bacterial pathogen infections in the intestinal epithelium. This means that CN therapy could aid in the development of new therapeutic strategies to control bacterial infections without treatment with anti-biotics, ultimately providing deeper insight into various intestinal disorders.

## 5. Conclusions

Overall, these findings highlight the relevance of the bioactivity of CN in the gut to block the mitochondrial apoptotic signaling pathway induced by *V. vulnificus* VvhA and provide important insight into the potential for the development of therapeutic strategies and agents for *V. vulnificus* infections. Although our study demonstrated that CN significantly improves gastrointestinal functions during *V. vulnificus* infections, further research is required to establish in greater detail the effects of CN on gastrointestinal homeostasis, the microbiome, and on the mucosal immune system.

## Figures and Tables

**Table 1:** Plasmids and bacterial strains used in this study.

Strain or Plasmid	Relevant Haracteristics ^a^	Reference or Source
Bacterial strains		
*V. vulnificus*		
M06-24/O	Clinical isolate; virulent; WT	Laboratory collection
CMM111	M06-24/O vvhA:Pks1201; elastase deficient; vvhA mutant	[15]
*E. coli*		
BL21 (DE3)	F^−^ *ompT hsdS_B_* (r_B_^−^m_B_^−^) *gal dcm* (DE3)	Laboratory collection
Plasmids		
pET29a (+)	His_6_ tag fusion expression vector; Km^r^	Novagen
pKS1201	pET29a (+) with VvhBA; Km^r^	This study

^a^ Km^r^, kanamycin resistan.

## Abbreviations:

Bax: Bcl-2 associated X protein; CM-H_2_DCFDA: 2′, 7′-dichlorofluorescein diacetate; CN: Curcumin nanosphere; FE-SEM: Field emission scanning electron microscope; JNK: c-Jun *n*-terminal kinase; MAPKs: Mitogen-activated protein kinases; NAC, *n*-acetyl-L-cysteine; NOX2: NADPH oxidase 2; NF-κB: Nuclear factor-kappa B; PKC: Phosphorylation of protein kinase C; PVDF: Polyvinylidene fluoride; ROS: Reactive oxygen species; SDS-PAGE: Sodium dodecyl sulfate polyacrylamide gel electrophoresis; TUNEL: Terminal deoxynucleotidyl transferase dUTP nick end labeling; *V. vulnificus*: *Vibrio vulnificus.*

## References

[1] Li G., Wang M.Y. (2019). The role of *Vibrio vulnificus* virulence factors and regulators in its infection-induced sepsis. Folia Microbiol..

[2] Song E.J., Lee S.J., Lim H.S., Kim J.S., Jang K.K., Choi S.H., Han H.J. (2016). *Vibrio vulnificus* VvhA induces autophagy-related cell death through the lipid raft-dependent c-Src/NOX signaling pathway. Sci. Rep..

[3] Jeong H.G., Satchell K.J. (2012). Additive function of *Vibrio vulnificus* MARTX_Vv_ and VvhA cytolysins promotes rapid growth and epithelial tissue necrosis during intestinal infection. PLoS Pathog..

[4] Lee Y.M., Park J.P., Lim K.T., Lee S.J. (2019). Intestinal epithelial cell apoptosis due to a hemolytic toxin from *Vibrio vulnificus* and protection by a 36kDa glycoprotein from *Rhus verniciflua* Stokes. Food Chem. Toxicol..

[5] Lee S.J., Jung Y.H., Song E.J., Jang K.K., Choi S.H., Han H.J. (2015). *Vibrio vulnificus* VvpE stimulates IL-1β production by the hypomethylation of the IL-1β promoter and NF-κB activation via lipid raft-dependent ANXA2 recruitment and reactive oxygen species signaling in intestinal epithelial cells. J. Immunol..

[6] Lee Y.M., Park J.P., Jung Y.H., Lee H.J., Kim J.S., Choi G.E., Han H.J., Lee S.J. (2020). Melatonin restores Muc2 depletion induced by *V. vulnificus* VvpM via melatonin receptor 2 coupling with Gαq. J. Biomed. Sci..

[7] Lee S.J., Lee H.J., Jung Y.H., Kim J.S., Choi S.H., Han H.J. (2018). Melatonin inhibits apoptotic cell death induced by *Vibrio vulnificus* VvhA via melatonin receptor 2 coupling with NCF-1. Cell Death Dis..

[8] Liu Q., Meng X., Li Y., Zhao C.N., Tang G.Y., Li H.B. (2017). Antibacterial and antifungal activities of spices. Int. J. Mol. Sci..

[9] Goel A., Kunnumakkara A.B., Aggarwal B.B. (2008). Curcumin as “curecumin”: From kitchen to clinic. Biochem. Pharmacol..

[10] Dulbecco P., Savarino V. (2013). Therapeutic potential of curcumin in digestive diseases. World J. Gastroenterol..

[11] Gera M., Sharma N., Ghosh M., Huynh D.L., Lee S.J., Min T., Kwon T., Jeong D.K. (2017). Nanoformulations of curcumin: An emerging paradigm for improved remedial application. Oncotarget.

[12] Park J.B., Lee Y.M., Park M.K., Min T., Lee S.J. (2019). Effects of anti-ecotoxicological curcumin nanospheres on feed efficiency and fecal odor in mice. J. Environ. Sci. Int..

[13] Ohno M., Nishida A., Sugitani Y., Nishino K., Inatomi O., Sugimoto M., Kawahara M., Andoh A. (2017). Nanoparticle curcumin ameliorates experimental colitis via modulation of gut microbiota and induction of regulatory T cells. PLoS ONE.

[14] Lee S.J., Jung Y.H., Oh S.Y., Jang K.K., Lee H.S., Choi S.H., Han H.J. (2015). *Vibrio vulnificus* VvpE inhibits mucin 2 expression by hypermethylation via lipid raft-mediated ROS signaling in intestinal epithelial cells. Cell Death Dis..

[15] Paramera E.I., Konteles S.J., Karathanosa V.T. (2011). Microencapsulation of curcumin in cells of *Saccharomyces cerevisiae*. Food Chem..

[16] Schmitter T., Pils S., Weibel S., Agerer F., Peterson L., Buntru A., Kopp K., Hauck C.R. (2007). Opa proteins of pathogenic neisseriae initiate Src kinase-dependent or lipid raft-mediated uptake via distinct human carcinoembryonic antigen-related cell adhesion molecule isoforms. Infect. Immun..

[17] Brandt D., Gimona M., Hillmann M., Haller H., Mischak H. (2002). Protein kinase C induces actin reorganization via a Src- and Rho-dependent pathway. J. Biol. Chem..

[18] Yousuf M.A., Lee J.S., Zhou X., Ramke M., Lee J.Y., Chodosh J., Rajaiya J. (2016). Protein kinase C signaling in adenoviral infection. Biochemistry.

[19] Jang B.C., Lim K.J., Paik J.H., Kwon Y.K., Shin S.W., Kim S.C., Jung T.Y., Kwon T.K., Cho J.W., Baek W.K. (2004). Up-regulation of human β-defensin 2 by interleukin-1β in A549 cells: Involvement of PI3K, PKC, p38 MAPK, JNK, and NF-κB. Biochem. Biophys. Res. Commun..

[20] Park K.A., Byun H.S., Won M., Yang K.J., Shin S., Piao L., Kim J.M., Yoon W.H., Junn E., Park J. (2007). Sustained activation of protein kinase C downregulates nuclear factor-κB signaling by dissociation of IKK-γ and Hsp90 complex in human colonic epithelial cells. Carcinogenesis.

[21] Baeuerle P.A., Baltimore D. (1996). NF-κB: Ten years after. Cell.

[22] Liu C.H., Chang F.Y. (2011). Development and characterization of eucalyptol microemulsions for topic delivery of curcumin. Chem. Pharm. Bull..

[23] Chen X., Zou L.Q., Niu J., Liu W., Peng S.F., Liu C.M. (2015). The stability, sustained release and cellular antioxidant activity of curcumin nanoliposomes. Molecules.

[24] Lee S.J., Jung Y.H., Oh S.Y., Song E.J., Choi S.H., Han H.J. (2015). *Vibrio vulnificus* VvhA induces NF-κB-dependent mitochondrial cell death via lipid raft-mediated ROS production in intestinal epithelial cells. Cell Death Dis..

[25] Kaul S., Blackford J.A., Cho S., Simons S.S. (2002). Ubc9 is a novel modulator of the induction properties of glucocorticoid receptors. J. Biol. Chem..

[26] Crane J.K., Vezina C.M. (2005). Externalization of host cell protein kinase C during enteropathogenic *Escherichia coli* infection. Cell Death Differ..

[27] Monturiol-Gross L., Flores-Diaz M., Pineda-Padilla M.J., Castro-Castro A.C., Alape-Giron A. (2014). *Clostridium perfringens* phospholipase C induced ROS production and cytotoxicity require PKC, MEK1 and NF-κB activation. PLoS ONE.

[28] Li J.D., Feng W., Gallup M., Kim J.H., Gum J., Kim Y., Basbaum C. (1998). Activation of NF-κB via a Src-dependent Ras-MAPK-pp90rsk pathway is required for *Pseudomonas aeruginosa*-induced mucin overproduction in epithelial cells. Proc. Natl. Acad. Sci. USA.

[29] Chen J.C., Wu M.L., Huang K.C., Lin W.W. (2008). HMG-CoA reductase inhibitors activate the unfolded protein response and induce cytoprotective GRP78 expression. Cardiovasc. Res..

[30] Ki M.R., Lee H.R., Goo M.J., Hong I.H., Do S.H., Jeong D.H., Yang H.J., Yuan D.W., Park J.K., Jeong K.S. (2008). Differential regulation of ERK1/2 and p38 MAP kinases in VacA-induced apoptosis of gastric epithelial cells. Am. J. Physiol. Gastrointest. Liver Physiol..

[31] Kohchi C., Inagawa H., Nishizawa T., Soma G. (2009). ROS and innate immunity. Anticancer Res..

[32] Rahman M.M., McFadden G. (2011). Modulation of NF-κB signalling by microbial pathogens. Nat. Rev. Microbiol..

[33] Mandal M., Olson D.J., Sharma T., Vadlamudi R.K., Kumar R. (2001). Butyric acid induces apoptosis by up-regulating Bax expression via stimulation of the c-Jun *n*-terminal kinase/activation protein-1 pathway in human colon cancer cells. Gastroenterology.

[34] Li P., Nijhawan D., Budihardjo I., Srinivasula S.M., Ahmad M., Alnemri E.S., Wang X. (1997). Cytochrome c and dATP-dependent formation of Apaf-1/caspase-9 complex initiates an apoptotic protease cascade. Cell.

[35] Sharpe J.C., Arnoult D., Youle R.J. (2004). Control of mitochondrial permeability by Bcl-2 family members. Biochim. Biophys. Acta.

[36] Hildebrand A., Pohl M., Bhakdi S. (1991). *Staphylococcus aureus* α-toxin. Dual mechanism of binding to target cells. J. Biol. Chem..

[37] Nougayrede J.P., Donnenberg M.S. (2004). Enteropathogenic *Escherichia coli* EspF is targeted to mitochondria and is required to initiate the mitochondrial death pathway. Cell. Microbiol..

[38] Jeong T.C., Kim H.J., Park J.I., Ha C.S., Park J.D., Kim S.I., Roh J.K. (1997). Protective effects of red ginseng saponins against carbon tetrachloride-induced hepatotoxicity in Sprague Dawley rats. Planta Med..

